# Probing Liver Injuries Induced by Thioacetamide in Human In Vitro Pooled Hepatocyte Experiments

**DOI:** 10.3390/ijms25063265

**Published:** 2024-03-13

**Authors:** Himanshu Goel, Richard L. Printz, Venkat R. Pannala, Mohamed Diwan M. AbdulHameed, Anders Wallqvist

**Affiliations:** 1 Department of Defense Biotechnology High Performance Computing Software Applications Institute, Telemedicine and Advanced Technology Research Center, U.S. Army Medical Research and Development Command, Fort Detrick, Frederick, MD 21702, USA; vpannala@bhsai.org (V.R.P.); mabdulhameed@bhsai.org (M.D.M.A.); 2 The Henry M. Jackson Foundation for the Advancement of Military Medicine, Inc., Bethesda, MD 20817, USA; 3 Department of Molecular Physiology and Biophysics, Vanderbilt University School of Medicine, Nashville, TN 37232, USA; r.printz@vumc.org; 4 Division of Diabetes, Endocrinology and Metabolism, Department of Medicine, Vanderbilt University Medical Center, Nashville, TN 37232, USA

**Keywords:** predictive toxicology, RNA-seq, thioacetamide, in vitro multi-donor pooled hepatocytes, toxicity modules, liver injury, KEGG pathways, toxicogenomics

## Abstract

Animal studies are typically utilized to understand the complex mechanisms associated with toxicant-induced hepatotoxicity. Among the alternative approaches to animal studies, in vitro pooled human hepatocytes have the potential to capture population variability. Here, we examined the effect of the hepatotoxicant thioacetamide on pooled human hepatocytes, divided into five lots, obtained from forty diverse donors. For 24 h, pooled human hepatocytes were exposed to vehicle, 1.33 mM (low dose), and 12 mM (high dose) thioacetamide, followed by RNA-seq analysis. We assessed gene expression variability using heat maps, correlation plots, and statistical variance. We used KEGG pathways and co-expression modules to identify underlying physiological processes/pathways. The co-expression module analysis showed that the majority of the lots exhibited activation for the bile duct proliferation module. Despite lot-to-lot variability, we identified a set of common differentially expressed genes across the lots with similarities in their response to amino acid, lipid, and carbohydrate metabolism. We also examined efflux transporters and found larger lot-to-lot variability in their expression patterns, indicating a potential for alteration in toxicant bioavailability within the cells, which could in turn affect the gene expression patterns between the lots. Overall, our analysis highlights the challenges in using pooled hepatocytes to understand mechanisms of toxicity.

## 1. Introduction

Exposure to toxic chemicals stemming from environmental conditions, contaminated food, or water can cause health concerns and may lead to acute or long-term injuries. Methods to evaluate toxicant-induced responses and their potential to cause adverse effects range from animal chemical exposure studies to different cell-based in vitro approaches. In vitro experiments provide the possibility of higher throughput investigations as compared to animal experimentation; however, the correlation between the in vivo and in vitro experiments as well as the interspecies correlation between the animal and human injury response remain challenging [1]. Primary animal hepatocytes have frequently been used to study liver function and disease mechanisms, and the primary hepatocyte culture is recognized as the most relevant in vitro model for studying hepatotoxicity and screening chemicals for drug-induced liver injuries. Primary human hepatocytes are thus considered the gold standard for qualitatively predicting the metabolism, toxicity, and drug interaction profiles of chemicals [2,3]. However, the results derived from human hepatocyte culture studies likely differ based on the demographic, genetic, and environmental characteristics of the donors from which the cells were cultured, as well as the techniques used to preserve the hepatocyte functionality outside of the body [4,5]. Overall, for a given toxicant, it is often challenging to identify common expression patterns from different in vitro studies conducted using hepatocytes cultured from independent donors. Hence, pooled hepatocyte cultures, obtained from multiple donors, offer the opportunity to reduce variability and devise a better workflow for consistently analyzing data from these studies.

The field of toxicogenomics has contributed to the understanding of how gene expression patterns and subsequent affected pathways may characterize the physiological response to drugs and toxicants [6]. A broad range of methods and platforms can map the connectivity between toxicant exposure and the affected genes. These methods enable the identification of molecular mechanisms, relevant biomarkers, phenotypic processes or injuries, and risk assessment profiles for a given toxicant. Our research group has used a variety of approaches to identify significantly expressed genes and their altered expression in order to assess the likely impact of exposure to a variety of toxicants (thioacetamide, acetaminophen, bromobenzene, carbon tetrachloride, and mercuric chloride) [7,8,9,10,11,12]. In particular, our earlier work on thioacetamide presented thorough toxicogenomic analyses of rat and guinea pig livers and kidneys, as well as of in vitro gene expression data from human hepatocytes [10]. The objective of the current study is to assess the responses of pooled human hepatocytes to thioacetamide exposure. Thioacetamide, which is known to cause liver injury, is often used as a model hepatotoxicant in a variety of experimental studies. Although previous studies have provided key insights, additional analyses of multi-donor pooled hepatocytes for liver injury are needed to validate the study outcomes and characterize toxicity-induced responses using toxicogenomic approaches.

Here, we sought to investigate the gene expression responses from the impact of thioacetamide exposure using RNA-seq data from multiple lots of pooled human hepatocytes. We evaluated five individual lots of pooled hepatocytes, and also evaluated the lots merged together. Each of the five lots contained primary hepatocytes that were obtained from either five or ten individual donors. The advantage of pooling hepatocytes from different donors is to minimize the variation that may arise from a comparison of hepatocytes derived from a single donor, thereby improving the predictability of gene expression outcomes. Thus, the current study aimed to analyze uniformity and variability in pooled hepatocyte cultures using different toxicogenomic approaches based on the information from gene level expression data, KEGG (Kyoto Encyclopedia of Genes and Genomes) pathways, and toxicity co-expression modules. Our multi-faceted approach will help elucidate the utility of different tools to express similarities and differences in pooled hepatocyte research. In addition, we analyzed the pattern for the key drug metabolism cytochrome P450 enzymes and hepatocyte transporters from the ABC and SLC families to demonstrate pooled hepatocyte lot-to-lot variability and uniformity. Some genes consistently showed up- or down-regulation patterns, such as *ATP8B1*, *ABCC2*, *CYP26A1*, *CYP26B1*, *CYP2C9*, and *CYP3A4/5*; however, a number of genes, such as *ABCB1*, *ABCC4*, *SLC22A7*, and *ABCB4*, showed significant fluctuation and opposite patterns across different lots.

## 2. Results and Discussion

### 2.1. Dose Optimization for Thioacetamide Exposure

To determine the dose of thioacetamide and the length of exposure needed to cause a detectable level of cell injury in a pool of primary human hepatocytes derived from multiple donors, we carried out a time-course/dose–response study using the two different following methods to detect injury: (1) we quantified intracellular ATP levels, and (2) we quantified lactate dehydrogenase activity in the media. Measuring cell viability based on metabolic activity, i.e., ATP levels, provided quality data for the determination of the optimal thioacetamide dose and the length of exposure with minimal variation among treatment replicates (see Figures S1–S3).

Figure S1 illustrates the time-course study to determine the amount of thioacetamide exposure needed to induce detectable cell injury in the pooled primary human hepatocytes. We tested eight doses (0, 0.049, 0.148, 0.444, 1.333, 4, 12, and 36 mM) with six different lengths of exposure (4, 8, 12, 16, 20, and 24 h) for a total of 48 conditions. We determined the minimum time to respond for each dose and observed a near-maximal reduction in cell viability occurring as early as 4 h, reaching a maximum by 16 h, and maintaining this maximum through 24 h. Figures S2 and S3 quantify cell viability versus the thioacetamide dose at 24 and 4 h of exposure, respectively. Figure S2 illustrates a dose-dependent decline in cell viability following thioacetamide treatment for 24 h, with doses ranging from 0.049 to 36 mM, as compared to a smaller reduction in cell viability after 4 h of exposure, shown in Figure S3. We determined that cell viability was dependent on exposure time and concluded that a 24 h exposure to thioacetamide was optimal for both a low dose (1.33 mM), which caused minimal cell injury, and a high dose (12 mM), which resulted in an easily detectable cell injury.

The second method used to measure cell viability, which assessed cell membrane integrity by measuring the activity of lactate dehydrogenase released into the culture medium after the thioacetamide treatment (see Figure S4), did not yield high-quality data, but did yield results that agreed with those obtained with the metabolic activity-based assay. Figure S4 shows a dose-dependent increase in lactate dehydrogenase activity in the culture medium after 24 h of exposure to various doses of thioacetamide, which indicates a reduction in cell membrane integrity and cell viability as the concentration of thioacetamide increased from 0.049 to 36 mM. These results support the conclusions regarding the optimal time and doses of thioacetamide needed to induce cell injury in pooled primary human hepatocytes.

**Figure 1:**
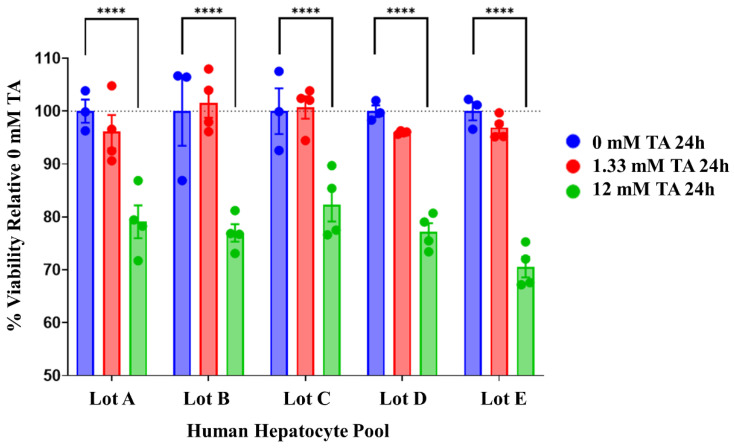
Measurement of cell viability for each lot of pooled human hepatocytes subjected to three different treatment conditions [one control and two doses of thioacetamide (TA)]. Lots A and B each contained pooled hepatocytes from five different donors, whereas each of the other lots are pools of hepatocytes derived from ten different donors. The star (****) symbol indicates *p*-values less than 0.05.

In addition to these preliminary tests, we assessed the efficacy of the low- and high-dose thioacetamide treatments by performing a cell viability assay on the same five lots of pooled primary human hepatocytes utilized for the isolation of RNA for our RNA-seq investigation of the gene expression responses following the thioacetamide exposure of five different lots of pooled human hepatocytes, labeled lots A–E. Lots A and B each consisted of a pool of human hepatocytes from five different donors, whereas each of the other lots consisted of a pool of hepatocytes derived from ten different donors. Figure 1 shows the cell viability assay results, which suggested a 2% reduction in viability with the low-dose (1.33 mM) treatment and a highly significant 33% reduction in viability with the high-dose (12 mM) treatment. Each lot of pooled hepatocytes responded similarly to 24 h of the thioacetamide treatment, thus suggesting that the cells treated for the isolation of RNA would likely have only a slight injury at the low dose and a significant injury at the high dose of thioacetamide. Given the outcomes of both doses, we focused our RNA-seq analysis on the cells exposed to the high dose of thioacetamide due to the detection of a significant reduction in cell viability as compared to the lower dose.

### 2.2. Gene-Level Analysis

**Figure 2:**
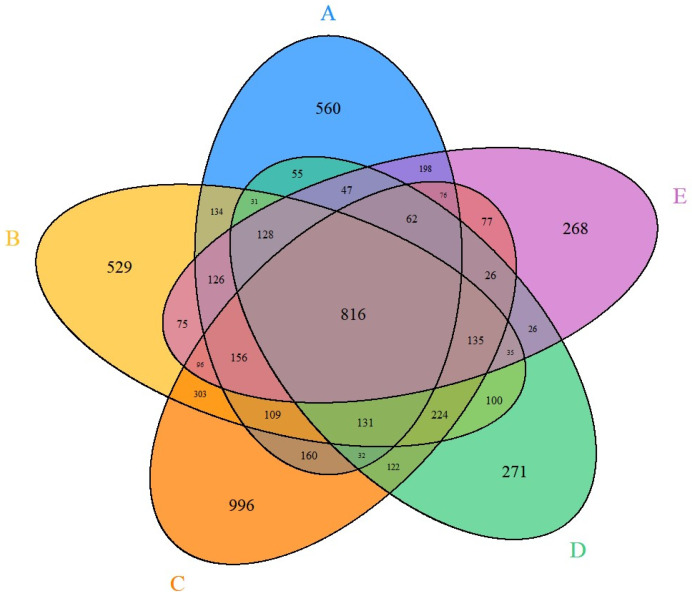
Venn diagram depicting the common differentially expressed genes across different lots (A–E) of in vitro pooled human hepatocytes exposed to thioacetamide (12 mM) treatment.

The RNA-seq data provide the individual gene expression responses induced by the toxicant exposure. Table 1 indicates in bold text the number of expressed genes whose expression was altered significantly after different lots (A–E) of pooled primary human hepatocytes were exposed for 24 h to thioacetamide at a dose of 12 mM. In addition, the number of differentially expressed genes found to be common between the different lots of pooled primary human hepatocytes are indicated in Table 1 as plain text. We identified a large lot-to-lot variability in the number of significantly altered genes, ranging from 2241 to 3521. This large variability highlights the variation seen across different lots of cells derived from multiple donors. However, the presence of a large number of gene overlaps between different lots also highlights the presence of similarities among genes affected by the thioacetamide toxicant. Additionally, we compared the differentially expressed genes across all lots and found 816 common genes (Figure 2). Identifying these overlapping genes allowed us to focus on the commonality of the involvement of these genes and their potential role in liver function and injury. Table 2 shows the top 10 up- and down-regulated genes from the 816 common gene set based on the average fold-change values from the lots. The down-regulation of the top two genes, *CYP26B1* and *CYP26A1*, is noteworthy because they are largely responsible for retinoic acid (RA) clearance in the liver, as it is metabolized to 1-oxo-RA and 4-OH-RA by these enzymes [13]. When rats were given thioacetamide, there was an observed rise in RA and a corresponding decrease in the activity of enzymes involved in retinoid metabolism [14]. Particularly, mRNA expression studies have examined *CYP26A1* mRNA isoforms in the human liver as a major contributor to RA clearance in the liver [15,16,17]. The metabolism of retinoid acid catabolism in the body is usually attributed to both of these genes [18,19]. Additionally, both of these genes have been highlighted in the work of Guo et al. [20], and are the most variable expressed genes among multiple donors [20]. On the other hand, the top two up-regulated genes, *C2CD4B* and *C2CD4A*, have multiple functional associations but are largely involved in the regulation of the inflammatory response [21]. The *SPP1* and *THRSP* genes have also been shown to be among the top 10 differentially expressed genes, in line with our previous research using thioacetamide liver injection in guinea pigs and rats [11]. In fact, *SPP1* is an important biomarker for fibrosis [22]. *FGF21* is another significant gene, and according to recent research, it plays a significant role in lipid metabolism and reduces hepatocyte damage [23,24,25,26,27]. Moreover, the activation of several of these genes, such as *SPP1*, *THRSP*, *SLCO4C1*, *SERPINA1*, and *DDAH2*, is well known and associated with liver injuries [28,29,30,31,32]. Additional up- or down-regulated genes in Table 2 may be possible markers for early stage liver injury.

**Figure 3:**
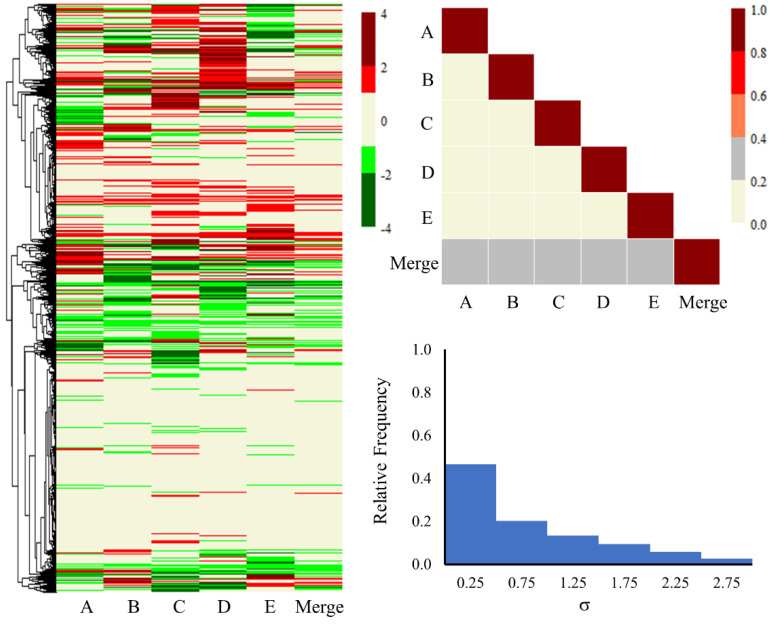
Heat map, correlation plot, and standard deviation histogram plot of all the genes obtained from each lot of pooled human hepatocytes exposed to thioacetamide as well as the merged set. The fold-change values ranging from −0.6 to 0.6 are shown in beige on the heat map. Significantly lower and higher values are shown in red and green, respectively.

**Figure 4:**
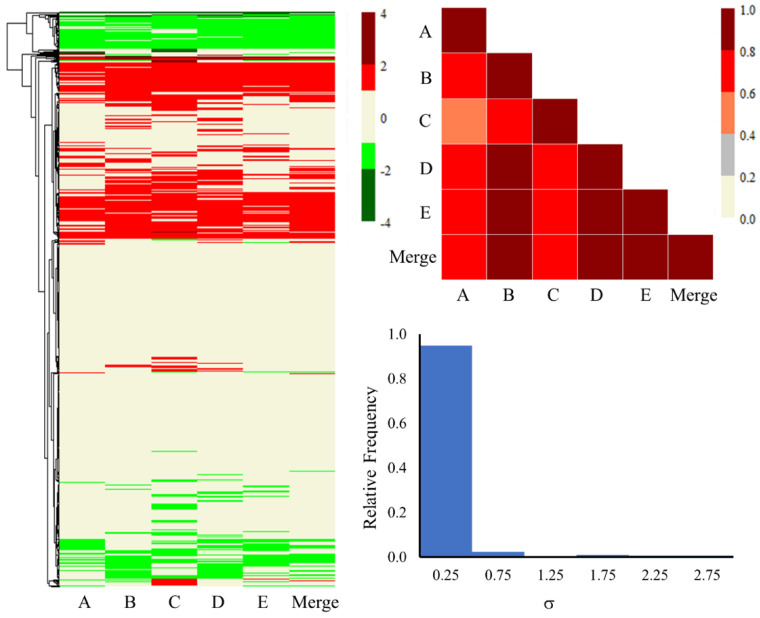
Heat map, correlation plot, and standard deviation histogram plot for the common 816 genes obtained from each lot of pooled human hepatocytes exposed to thioacetamide as well as the merged set. The fold-change values ranging from −0.6 to 0.6 are shown in beige on the heat map. Significantly lower and higher values are shown in red and green, respectively.

Furthermore, Figure 3 and Figure 4 illustrate the heat maps, correlation plots, and standard deviation (SD) histogram plots for all the genes, as well as the common 816 genes for each lot and the merged set. The merged column in the figures represents the average of the fold-change values of all five lots of primary human hepatocytes. Visualizing the expression of all the genes through heat maps and a triangle correlation matrix (Figure 3), one can observe relatively lower correlation scores (<0.20) from lot to lot, with some improvement (between 0.20 to 0.40) when compared to the merged dataset. The SD values were also slightly higher for several genes and were largely evenly distributed. We expected the lower correlation scores and larger SD values due to the involvement of roughly 15,000 genes and the wide demographic diversity of the donor hepatocytes. On the other hand, the heat map for the common 816 genes (Figure 4) is more clustered and homogeneous in up- and down-regulated gene expression profiles. Quantitatively, this can be observed in the higher correlation scores, varying from 0.55 to 0.89 between the different lots. Likewise, the SD histogram plot values for the common 816 genes (Figure 4) are below 0.50 for 95% of the genes. Overall, the lower SD values and strong correlation scores are encouraging and build confidence in the shared differentially expressed genes among the different lots. All the genes and their fold-change values from each lot and the merged lots can be found in the Supplementary Materials (Tables S1 and S2).

### 2.3. KEGG Pathway Analysis

The Kyoto Encyclopedia of Genes and Genomes (KEGG) pathway analysis is commonly used in bioinformatics to understand the biological function, signaling pathways, inflammatory responses, and molecular mechanisms based on the co-expressed genes list. Table 3 shows the 15 most enriched KEGG pathways that are common in all the lots for the thioacetamide experiments, with steroid biosynthesis at the top of the list. Our pathway analysis shows that most of the overlapping genes are associated with lipid, amino acid, carbohydrate, terpenoid, and polyketide metabolism. Lipid molecules are crucial in metabolic processes, and their alteration can lead to liver disease. As the liver is a site for the elimination of toxic metabolites, the peroxisome is involved in the biosynthesis of bile acids [33,34,35]. The p53 signaling pathway is also associated with liver inflammation, fibrosis, and steatosis, and its effect on the liver changes substantially at different stages of liver injury [36]. Furthermore, several other pathways listed in Table 3, such as tight junction and ribosome biogenesis in eukaryotes, are also reported to be associated with liver disease [37,38,39]. Collectively, these pathways show the potential impact of thioacetamide on the liver and how this can affect most of the metabolic pathways, leading to liver disorders.

**Figure 5:**
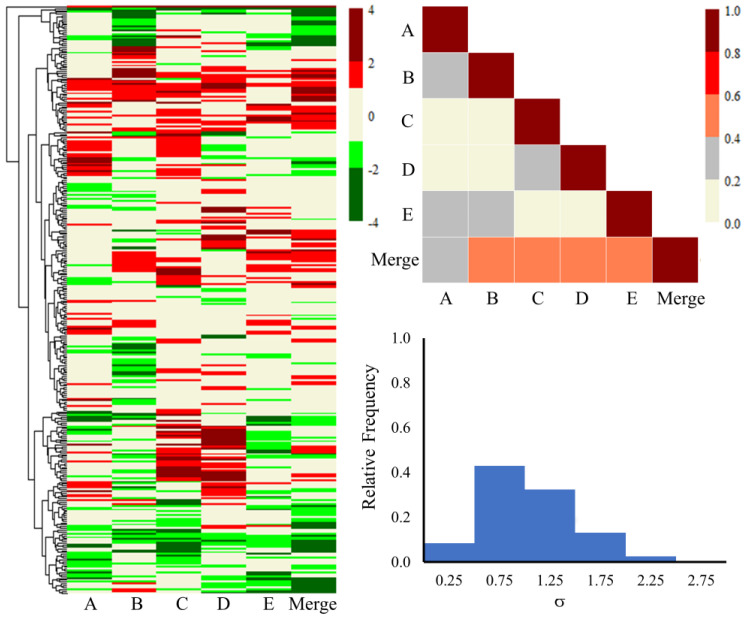
Heat map, correlation plot, and standard deviation histogram plot for the KEGG pathways using all the genes obtained from each individual lot of pooled human hepatocytes exposed to thioacetamide as well as the merged set. The z-score values ranging from −0.6 to 0.6 are shown in beige on the heat map. Significantly up- and down-regulated pathways are indicated in red and green, respectively.

**Figure 6:**
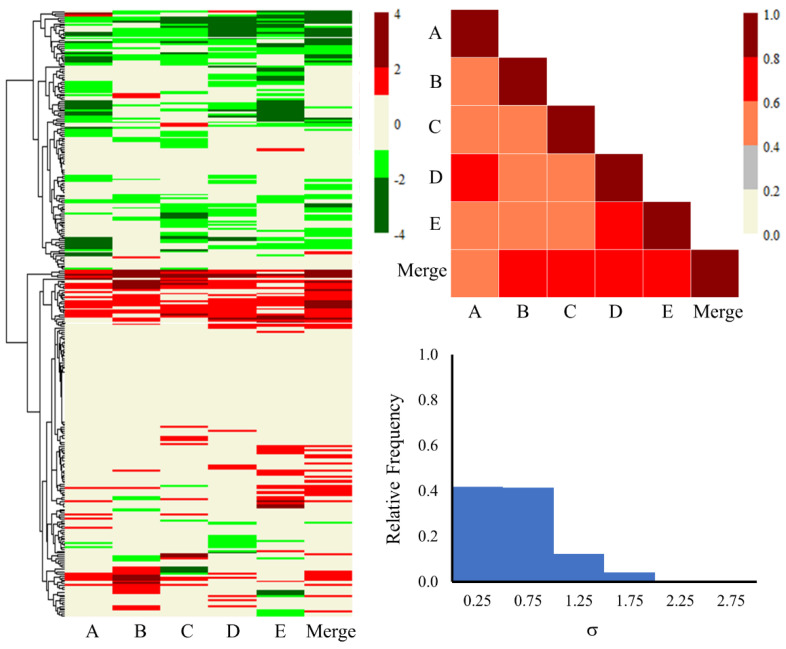
Heat map, correlation plot, and standard deviation histogram plot for the KEGG pathways using the common 816 genes obtained from each individual lot of pooled human hepatocytes exposed to thioacetamide as well as the merged set. The z-score values ranging from −0.6 to 0.6 are shown in beige on the heat map. Significantly up- and down-regulated pathways are indicated in red and green, respectively.

Additionally, Figure 5 shows the heat map, triangular correlation plot, and SD histogram plot for the KEGG pathways of all the genes for each lot and the merged set. Similarly, Figure 6 depicts the heat map, triangular correlation plot, and SD histogram plot for the KEGG pathways of the common 816 genes and their corresponding fold-change values for each lot and the merged set. The merged column in Figure 5 and Figure 6 represents the averaged z-score values of all five lots. The heat maps of the up- and down-regulated pathways, estimated with TOXPANEL version 1.0 [40], are shown in red and green, respectively. We provide detailed information for all KEGG pathways as well as their calculated scores for Figure 5 and Figure 6 in the Supplementary Materials (Tables S3 and S4). The heat maps created using the common genes show grouping of up- or down-regulated pathways and are more structured when compared to the pathway heat maps using all the genes. The majority of the metabolic pathways consistently exhibit up-regulation on the heat maps, while the signaling and cellular pathways exhibit down-regulation. In the following paragraph, we describe in depth the functional behavior of different pathways. The KEGG pathway correlation scores and the SD variations improved significantly in the pathways computed using the common 816 genes as compared to the results obtained from all the genes. The correlation scores between lots were below 0.40; these also improved (between 0.40 and 0.60) when linked with the merged lot. On the other hand, the correlation scores ranged from 0.40 to 0.80 for pathways obtained using the common genes. Likewise, 80% of the pathways had SD statistics below 1 using the common genes as compared to 50% when considering all the genes. In summary, the KEGG pathways using common differentially expressed genes revealed a consistent pattern of pathways underlying liver injury mechanisms.

**Figure 7:**
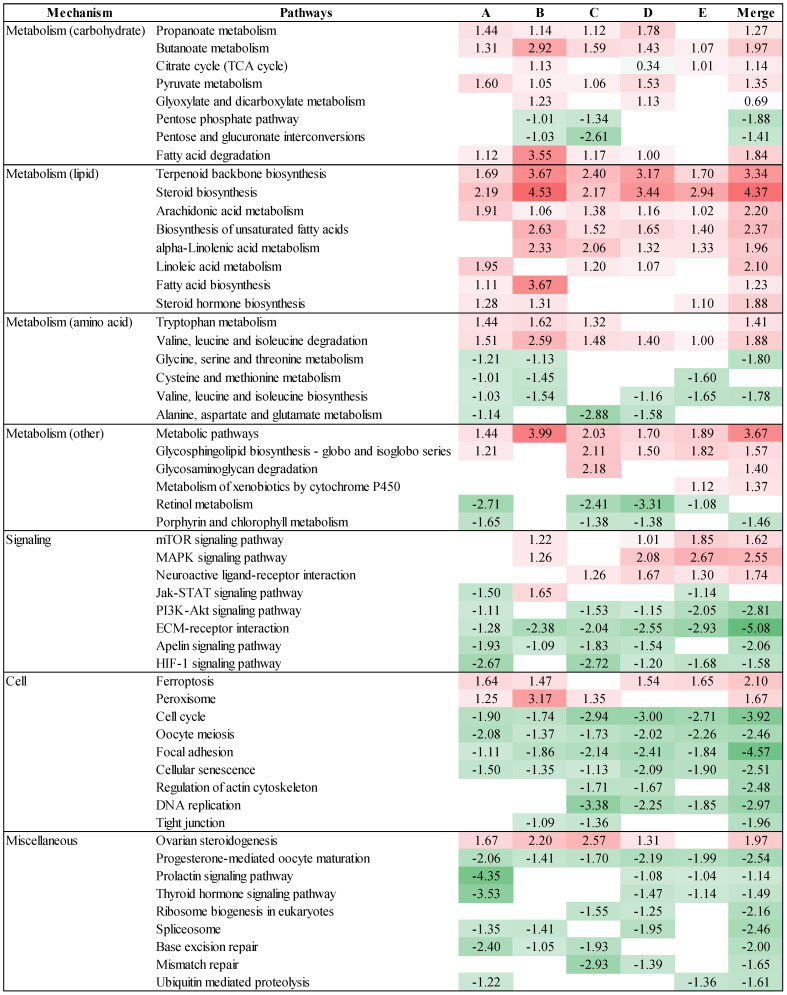
KEGG pathways activated using gene expression data from each individual lot of pooled human hepatocytes exposed to thioacetamide as well as the merged set. Significantly up- and down-regulated pathways are indicated in red and green, respectively.

A range of metabolic and signaling pathways as well as several cellular and inflammation processes are involved in the development of liver injury. Because of the diversity in pooled hepatocyte data, it is of the utmost importance to identify shared processes and pathways in order to better understand the molecular mechanisms. Figure 7 illustrates all the significant KEGG pathways identified in the in vitro pooled hepatocytes by employing the common 816 genes from all the individual lots and the merged set. We have categorized different pathways into groups, i.e., *Carbohydrate/Lipid/Amino acid/Other Metabolism*, *Signaling*, *Cellular*, and *Miscellaneous*. Every relevant pathway that is up-regulated or down-regulated can be identified by a color-coded z-score which corresponds to each lot. We found that thioacetamide exposure in pooled hepatocytes altered several metabolic, signaling, and cellular response pathways. Figure 7 shows a substantial elevation in important metabolic pathways associated with lipid, amino acid, carbohydrate, and other metabolisms. Specifically, all lots exhibited elevated z-scores in the lipid metabolic pathways involving arachidonic acid metabolism, terpenoid backbone biosynthesis, and steroid biosynthesis. The enrichment analysis also demonstrated the importance of the metabolic pathways and the production of terpenoid backbone and steroids biosynthesis pathways. We observed very similar responses when we exposed the primary hepatocyte cells in our earlier in vitro human cells study to thioacetamide-S-oxide, a metabolite form of thioacetamide [10]. Furthermore, our group’s other toxicity and metabolomics studies involving thioacetamide-induced injury in guinea pigs and rats demonstrated up-regulated fold-change values for the metabolism of lipids and amino acids [11].

Several studies in the literature indicate that thioacetamide treatment also activates the mitogen-activated protein kinase (MAPK) pathway in liver tissue, and our data demonstrate significant alterations in this pathway in multiple lots as well [41]. We observed a substantial down-regulation of the apelin, hypoxia-inducible factor (HIF)-1, and phosphatidylinositol 3-kinase (PI3K)-protein kinase B (AKT) signaling pathways, in addition to extracellular matrix (ECM)–receptor interactions. Our results show a considerable change in the alteration of ECM interactions, which has been linked to the imbalance and impairment of liver function upon treatment with thioacetamide [42,43]. Liver disease progression is known to be related to HIF, PI3K-AKT, and apelin pathways, which are known to participate in different cell activations of inflammation and proliferation [44,45,46]. A number of other cellular and miscellaneous pathways also showed consistent down-regulation trends across all the lots after thioacetamide exposure. These pathways include cell cycle, oocyte meiosis, focal adhesion and cellular senescence, DNA replication, base excision repair, and progesterone-mediated oocyte maturation.

### 2.4. Liver Injury Module Analysis

**Figure 8:**
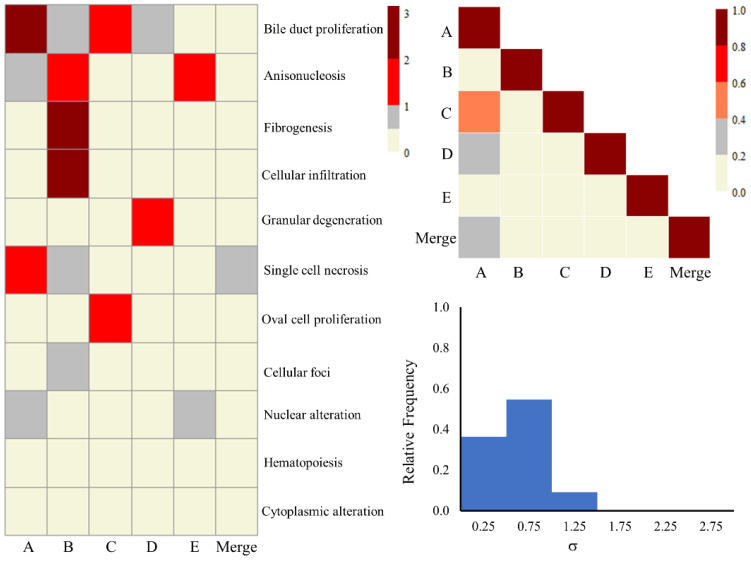
Heat map, correlation plot, and standard deviation histogram plot (for lots A to E) for liver injury modules obtained using the gene expression data from each individual lot of pooled human hepatocytes exposed to thioacetamide as well as the merged set.

Our research group has formulated eight kidney injury and eleven liver injury modules involving several sets of genes linked with histopathological injury phenotypes [40]. Each module indicates a set of co-expressed genes representing a well-defined histopathological injury endpoint. This analysis is available via the TOXPANEL software [40]. We analyzed the responses of these co-expressed genes to assess the impact of liver injury in different lots. Figure 8 shows the heat map for liver injury module scores in all the lots after exposure, as well as the correlation scores and SD histogram. We observed weaker activation for most of the liver injury modules and large variability in the fold-change-based z-score metric. We mainly identified the activation of bile duct proliferation and anisonucleosis injury modules in some of the lots following thioacetamide exposure. Other modules only occasionally showed activation in one of the lots. The activation of the bile duct proliferation module is commonly associated with a ductular reaction and results in acute or end-stage chronic liver disorders in patients and animal models [47,48,49,50]. We also observed weaker activation scores for all the modules in our previous study of the thioacetamide-S-oxide treatment of human single-donor primary hepatocytes [10]. While the earlier study involved a toxicant in the form of a metabolite of thioacetamide and the dosage was different from this study, the pattern of lesser activation scores was still visible. Notably, the bile duct proliferation module was also activated in the human single-donor primary hepatocyte study [10]. In our earlier work, we established an appropriate threshold value of 2 for the z-score metric in order to classify the degree of activation for a module representing a significant indication of injury [40]. However, we saw weaker z-scores in the case of in vitro single-donor human primary or pooled hepatocytes when compared to other in vivo or in vitro animal experiments. The inter-lot correlation also produced lower correlation scores (below 0.2) due to the excessive variation of the module’s z-scores, with a few exceptions. Lot comparisons A to C and A to D showed correlation scores of 0.50 and 0.30, respectively. The SD histogram also reflected large SD values, given the weaker z-scores for most of the liver injury modules. We provide all the z-score values for all the modules in the Supplementary Materials (Table S5). Furthermore, to understand the variations in the lots, we analyzed three different liver toxicity gene sets that contained co-expression genes associated with liver toxicity. Table 4 shows the performance of the predictive toxicogenomics space (PTGS) (all) [51], PTGS (core) [51], and toxicity module gene (TMG) [52,53] for all the lots subjected to the thioacetamide exposure. For each feature set, we calculated the absolute average fold-change values of the co-expressed genes belonging to each gene set. The variability among the different lots ranged from 0.75 to 0.94 for PTGS (all), 0.71 to 0.89 for PTGS (core), and 0.68 to 0.83 for TMG. The average (SD) values for each gene set’s scores across all the lots were 0.84(0.07), 0.80(0.06), and 0.75(0.06) for PTGS (all), PTGS (core), and TMG, respectively. We found an overall variability of ~10% for each gene set across the different lots, which derives from the differences in pooled cultures found between the lots.

### 2.5. Hepatocyte Transporters and Cytochrome P450 Enzymes

The primary organ in charge of metabolism is the liver, where transporters play a role in managing uptake and efflux. Alterations in hepatocyte transporters and cytochrome P450 (CYP) enzymes can contribute to liver injury. Table 5 shows the main hepatocyte transporters from the ABC and SLC families that exhibited substantial fold-change values in one or more lots, as well as the average and SD values. The basolateral uptake transporter OATPs (organic anion-transporting polypeptides), OATs (organic anion transporters), and OCTs (organic cation transporters) are most frequently expressed in the liver and mediate the bidirectional transport of a wide variety of substances and molecules [31,54,55]. The basolateral transporter genes *SLCO1B3*, *SLCO2B1*, *SLC22A7*, and *SLC22A1* exhibited both up- and down-regulated patterns from various lots, as well as significant fluctuations. OAT7 (*SLC22A9*) displayed fluctuations and indicated the constant up-regulation of the fold-change values across all the lots. Additionally, MRP3 and MRP4 are basolateral efflux transporters that transport large sets of endogenous and xenobiotic organic anions [54]. Both transporters showed substantial variations from lot to lot, and no inference can be made.

Bile acids and their salts are toxic to hepatocytes; hence, they must be effectively eliminated. The MDR3 transporter (phosphatidylcholine floppase, *ABCB4*) and P4-ATPase (*ATP8B1*) genes are ATP-binding cassette members and are crucial for the efflux of bile’s constituents [31,54,55,56,57]. We observed large fluctuations and an inconsistent pattern of up- or down-regulation for the MDR3 transporter across different lots. *ATP8B1*, another important hepatocyte transporter for the production of bile, prevents bile salt toxicity and functions as a phosphatidylserine translocase or flippase [58]. Table 5 shows a steady down-regulation pattern for this gene across all the lots. A deficiency of the *ATP8B1* transporter could result in irregular lipid composition in the membrane bilayer, causing bile acids to affect the function of membrane proteins and the bile salt export pump [58,59,60]. A loss of function of the *ATP8B1* gene could manifest clinically as progressive familial intrahepatic cholestasis type 1 (PFIC1), also known as Byler’s disease [58,59,60]. Furthermore, other canalicular efflux transporters, such as P-glycoprotein (Pgp, gene symbol *ABCB1*), breast cancer resistance protein (BCRP, gene symbol *ABCG2*), and multidrug resistance-associated protein 2 (MRP2, gene symbol *ABCC2*), are primarily responsible for multidrug resistance and eliminating bile from the hepatocytes [31,54,55]. All of the lots showed consistent up-regulated patterns for *ABCC2* and *ABCG2*; however, none of the lots had substantial fold-change values. We also saw a similar pattern with Pgp (*ABCB1*), with the exception of one lot, and lot E reported a fold-change value of 5.27. Additional SLC transporters (*SLC16A11* and *SLC16A13*), which mediate the influx and efflux of ions, nucleotides, and sugars, showed the consistent up-regulation of fold-change values and have been linked to liver injury [32].

The CYP family, which is largely expressed in the liver, is involved in the metabolism of drugs, chemicals, and endogenous substrates, and is crucial for the removal of various substances from the bloodstream [61,62]. Table 6 lists the prominent CYP enzymes from various lots, as well as their average and SD fold-change values. Except for a small number that displayed an opposite inclination, the majority of the major CYP enzymes showed a consistent pattern of up- or down-regulation throughout the various lots. The major CYP isoforms, namely, *CYP2C9*, *CYP2C19*, and *CYP3A4/5*, have the highest impact on drug metabolism and showed consistent up-regulation [63]. In particular, as described above, *CYP26A1* and *CYP26B1* had fold-change activities with a greater degree of down-regulation than any other enzymes. These CYP enzymes, thought to be involved in all-trans RA clearance, are members of the CYP26 family, including *CYP26A1* and *CYP26B1* [17,64]. Targeting RA signaling has been shown to be beneficial for humans, and numerous studies have demonstrated a relationship between the imbalance of RA and various liver diseases [13,15,16,65,66,67,68,69]. The *CYP1A1* enzyme plays a significant role in the conversion of procarcinogens into reactive metabolites and displayed a stronger up-regulation in four out of the five lots. The CYP2E1 enzymes, which cause oxidative stress and encourage the development of liver injury, also showed a less pronounced but persistently up-regulated pattern [70,71,72]. The CYP2E1 enzymes are also involved in the biotransformation of thioacetamide to thioacetamide-S-oxide/S-dioxide [42,73]. Many published studies have explicitly shown an inter-individual variability pattern for major drug-metabolizing CYP enzymes among different donors [63,74,75,76]. These results imply a heterogeneity of CYP expression across various donor liver pools. The CYP enzymes go through a complicated process that might differ greatly from donor to donor in terms of their expressions and mechanisms, and genetic testing may help clinicians diagnose and assess liver injury. Overall, it is essential to monitor changes in transporters and CYP enzymes in order to detect early indications of liver injury.

## 3. Materials and Methods

### 3.1. Chemicals and Reagents

We obtained multi-donor pools of plateable cryopreserved human hepatocytes (HPCH05+ and HPCH10+) in five different lots (2110283, 1810126, 1810050, 2010236, and 2210146) from a commercial vendor (Sekisui XenoTech, Kansas City, KS, USA), along with OptiThaw Hepatocyte Kit (K8000), OptiPlate Hepatocyte Media (K8200), and OptiCulture Hepatocyte Media (K8300) for culturing the hepatocytes. We secured Corning BioCoat 96-well clear (354407), 96-well white (354519), and 6-well clear (354400) collagen 1-coated plates from Thermo Fisher Scientific (Waltham, MA, USA). We purchased thioacetamide (163678) and a Lactate Dehydrogenase Activity Assay Kit (MAK066) from Sigma-Aldrich (St. Louis, MO, USA). We obtained a CellTiter-Glo 2.0 Cell Viability Assay (G9242) from Promega (Madison, WI, USA). We accomplished hepatocyte RNA isolation using the RNeasy Plus Mini Kit (74134) and RNase Free DNase Set (79254) from Qiagen (Germantown, MD, USA).

### 3.2. Cell Viability Assays

In order to optimize the dose and length of thioacetamide exposure needed to induce liver cell injury, we used a single pool of cells derived from 30 individual liver donors (15 males and 15 females) and prepared them by combining aliquots from four of the five lots (2110283, 1810126, 1810050, 2010236) of multi-donor pooled primary human hepatocytes (Sekisui XenoTech). We plated the hepatocytes from this single pool at a density of 40,000 cells per well in either white 96-well collagen 1-coated plates for the measurement of cell viability (intracellular ATP levels) after exposure to thioacetamide using the CellTiter-Glo 2.0 Cell Viability Assay (Promega) or in clear 96-well collagen 1-coated plates to measure cell viability (membrane integrity) using the Lactate Dehydrogenase Activity Assay Kit (Sigma-Aldrich). We thawed cryopreserved human hepatocytes and plated them following the supplier’s (Sekisui XenoTech) protocol using the cell culture reagents provided, OptiThaw Hepatocyte Kit (K8000), OptiPlate Hepatocyte Media (K8200), and OptiCulture Hepatocyte Media (K8300). The dosing of thioacetamide in OptiCulture Hepatocyte Media began 24 h after the hepatocytes were placed into culture. We tested 8 doses (0, 0.049, 0.15, 0.44, 1.33, 4.0, 12, and 36 mM) with 6 different lengths of exposure (4, 8, 12, 16, 20, and 24 h) for a total of 48 conditions. We plated the hepatocytes at a density of 40,000 cells per well in white 96-well collagen 1-coated plates following the supplier’s (Sekisui XenoTech) protocol using the cell culture reagents provided to assess the efficacy of the low-dose (1.33 mM) and high-dose (12 mM) thioacetamide treatments after 24 h on the same five lots of pooled primary human hepatocytes that were utilized to isolate RNA for RNA-seq analysis. We assessed the cell viability after the thioacetamide treatment using the CellTiter-Glo 2.0 Cell Viability Assay (Promega).

### 3.3. Experimental Procedures

**Figure 9:**
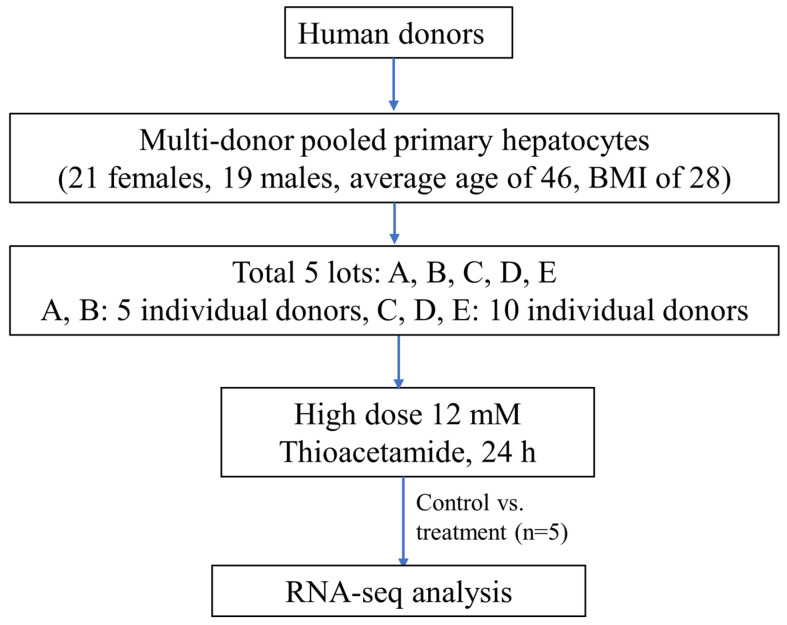
Experimental protocol for thioacetamide exposure to five lots of pooled human hepatocytes. BMI, body mass index.

We identified pools of plateable cryopreserved human hepatocytes, derived from multiple human liver donors, from a commercial vendor (Sekisui XenoTech). We obtained the metabolically characterized plateable hepatocytes as separate collections (lots) composed of groups of ten or five liver donors. We selected five different lots of multi-donor pooled primary human hepatocytes as follows: two lots (A: 2110283 and B: 1810126) consisted of pools from five individual donors, and three lots (C: 1810050, D: 2010236, and E: 2210146) consisted of pools from ten individual donors. The multi-donor pooled primary human hepatocytes were derived from 40 individual donors (39 unique donors), 21 females and 19 males, with an average age of 46 and body mass index of 28 for both males and females (Figure 9). We identified one donor, H1442, in two lots of pooled primary human hepatocytes, and indicated this in Figure S5, which shows a summary of the demographics of the 40 donors, varying in race, ethnicity, gender, and age, for the five lots of pooled primary human hepatocytes.

We treated the five different lots of multi-donor pools of primary human hepatocytes for 24 h with zero (control), 1.33 mM (low dose), and 12 mM (high dose) thioacetamide (MilliporeSigma, Burlington, MA, USA), with 1.33 mM and 12 mM representing the optimized low and high doses, respectively, to generate a minimal to a significant toxicant injury. These cell treatments generated a total of seventy-five RNA samples, five samples per each of the three treatments of the five different lots of liver cells. We plated and cultured each lot of pooled primary human hepatocytes into five wells of six-well, collagen 1-coated plates (Corning BioCoat Plates, Thermo Fisher Scientific) for each treatment condition using the protocols and reagents provided by the supplier (Sekisui XenoTech). We performed three treatments (0, 1.33, and 12 mM thioacetamide), thus, fifteen six-well plates with ~300,000 cells per well were used to isolate RNA samples from seventy-five wells of cells after 24 h of treatment. We isolated and purified total RNA using RNeasy Plus Mini Kits with in-column DNase I digestion (Qiagen) from these 75 samples and submitted all 75 RNA samples to the Vanderbilt University Medical Center VANTAGE Core (Nashville, TN, USA) for RNA sequencing. A stranded mRNA (polyA-selected) library preparation kit was used for mRNA enrichment and cDNA library production on each RNA sample. Sequencing was performed at the paired-end 150 bp on an Illumina NovaSeq 6000 (San Diego, CA, USA), targeting an average of 50 million reads per sample. Quality control reports for the sequencing and the per sample yield were provided. The RNA sequencing results were in the form of de-multiplexed FASTQ files. All the files obtained from the RNA-seq were placed in the NCBI’s Gene Expression Omnibus (GEO) database, using the GEO series accession number GSE250139.

### 3.4. Computational Details for RNA-Seq Data, KEGG Pathways, and Injury Module Analysis

We employed Kallisto [77,78] for the analysis of the multi-donor pooled human hepatocyte RNA-seq data. The program pseudo-aligns the reads to a reference and produces the quantification of the transcript abundances for each read. For this study, we downloaded the *Homo sapiens* transcriptome from the Ensemble [79] website and used it for the pseudo-aligning of the reads. We accomplished the estimation for the uncertainty of transcript abundance by repeating the analyses 100 times after resampling with replacement through the bootstrap technique. We used another related tool named Sleuth [80] for the analysis and comparison of all the transcript data, estimating the gene variance and leading to the identification of the significantly expressed genes. We set the criteria for significantly expressed genes with a false discovery rate-adjusted *p*-value (q-value) as ≤0.1.

Next, we used the publicly available gene co-expression-based tool TOXPANEL [40], formulated by our research group, to assess injury modules and KEGG pathways. This tool uses the gene expression of a set of genes to predict an adverse effect on the kidney or liver. Because it is challenging to predict organ injury or damaging effects based on individual genes, using the co-expressed gene set may be able to provide a clearer injury endpoint and mechanism. TOXPANEL uses the following two methods: aggregated absolute fold change (AAFC) and aggregated fold change (AFC) [7,8]. We input into TOXPANEL the responses from the set of genes, in the form of log-transformed gene expression values for the control and treatment groups. The AAFC method calculated the fold-change values for each gene by taking the difference between the control and treatment cohorts. The fold-change values for the set of pre-defined genes were subsequently grouped together, resulting in the z-score of each module or a particular pathway. Each module encompassed several genes along with their fold-change values. The AAFC method accounted only for the change in the set of genes and did not categorize the up- or down-regulated pattern of the genes. The AFC method can support this particular functionality and provide the direction for the gene expression sets, which becomes advantageous for the analysis of KEGG pathways [81]. The significance of each module was also estimated by the *p*-value, which is defined as the probability of the score for randomly (10,000 times) selected fold-change values becoming greater than the actual module score [40]. A lower *p*-value (preferably <0.05) indicated the importance of a particular module from the different modules. For additional details on TOXPANEL and its methods, we refer the reader to our earlier articles [7,8,40]. In addition, we utilized the DAVID [82] tool for the set of 816 common differentially expressed genes in order to perform the gene-enrichment/KEGG analysis.

## 4. Conclusions

Utilizing thioacetamide as a model hepatotoxicant, we collected transcriptomics data from multi-donor human pooled hepatocytes. We used five biological replicates (experimental lots) of pooled human hepatocytes, collected from several donors with different racial, gender, and age distributions, at two different thioacetamide concentrations. In this work, we focused on the high dose of thioacetamide as it led to a significant reduction in cell viability, thus likely generating an injury response. Based on the studies reported so far, the utility or suitability of pooled human hepatocytes to predict liver toxicity is unclear, and this motivated us to perform this study. We evaluated the generated gene expression data using conventional gene expression statistical methods, KEGG enrichment analysis to predict molecular pathways, and liver injury modules to predict histopathological outcomes as potential liver injury phenotypes. We identified significantly altered genes that displayed similarities in their response despite the high variability between the lots, and we found that several of these genes are associated with amino acid, lipid, and carbohydrate metabolism KEGG pathways. In particular, significant alterations in lipid metabolism pathways, including steroid, steroid hormone, and unsaturated fatty acid production, were evident and were associated with liver injuries. The pooled human hepatocytes showed weaker activation signals for most of the injury modules than previously observed with the in vitro single-donor primary hepatocytes; however, our analysis showed bile duct proliferation as a consistent change in the lot-to-lot readout. Despite the large inter-subject variability, we found a common set of differentially expressed genes that displayed similar behavior to thioacetamide across the lots of pooled human hepatocytes. This common gene set can be evaluated in future pooled hepatocyte experiments with hepatotoxicants. We also analyzed the expression pattern of key transporters and cytochrome P450 enzymes in the pooled human hepatocytes. We found larger lot-to-lot variability in their expression patterns, indicating a potential for alteration in toxicant bioavailability within the cells, which could in turn affect the gene expression patterns between the lots. Our work provides some key insights that should be considered in future pooled human hepatocyte experiments. Overall, the inherent variability of the characteristics of multi-donor pooled hepatocytes shows that additional work needs to be completed in order to capture and assess this variability and the impacts on toxicity studies.

## Figures and Tables

**Table 1:** Overlap of significantly altered differentially expressed genes (q < 0.1) found in human in vitro pooled hepatocyte data across five lots (A–E) for thioacetamide exposure.

Overlap	12 mM Thioacetamide				
Lots	A	B	C	D	E
A	**2821**	1631	1542	1302	1609
B		**3128**	1970	1600	1567
C			**3521**	1548	1444
D				**2241**	1275
E					**2347**

**Table 2:** Top 10 down- and up-regulated differentially expressed genes from the 816 common genes across all lots of pooled human hepatocyte and their average fold-change (FC) in expression and standard deviation (SD).

Gene (Down)	log_2_(FC)	SD	Gene (Up)	log_2_(FC)	SD
*CYP26B1*	−4.50	1.66	*C2CD4B*	2.42	0.39
*CYP26A1*	−3.75	2.00	*C2CD4A*	2.40	0.27
*IFNGR1*	−2.53	3.03	*SERPINA1*	2.32	4.92
*MCM10*	−2.15	0.78	*DDAH2*	2.08	2.17
*STEAP4*	−1.95	0.62	*THRSP*	1.85	0.52
*GNAO1*	−1.75	1.74	*FGF21*	1.81	0.22
*SPP1*	−1.44	0.28	*PIWIL2*	1.35	0.37
*LPCAT1*	−1.44	1.12	*FUT1*	1.32	0.23
*DTL*	−1.41	0.18	*SNORC*	1.26	0.08
*WDR76*	−1.33	0.23	*SLCO4C1*	1.21	0.35

**Table 3:** KEGG (Kyoto Encyclopedia of Genes and Genomes) pathways enriched in overlapping differentially expressed genes obtained from different lots of pooled primary hepatocytes in response to thioacetamide treatment.

KEGG Pathway (HD, 24 h)	Benjamini *p*-Value
Steroid biosynthesis	9.4 × 10^−6^
Peroxisome	6.5 × 10^−5^
Metabolic pathways	1.3 × 10^−3^
Carbon metabolism	4.8 × 10^−3^
Steroid hormone biosynthesis	8.4 × 10^−3^
Glyoxylate and dicarboxylate metabolism	9.2 × 10^−3^
Glycine, serine, and threonine metabolism	1.2 × 10^−2^
Biosynthesis of cofactors	1.2 × 10^−2^
Tight junction	1.6 × 10^−2^
p53 signaling pathway	2.0 × 10^−2^
Biosynthesis of amino acids	2.4 × 10^−2^
Terpenoid backbone biosynthesis	3.5 × 10^−2^
Ribosome biogenesis in eukaryotes	5.0 × 10^−2^
Biosynthesis of unsaturated fatty acids	7.0 × 10^−2^
DNA replication	7.0 × 10^−2^

**Table 4:** Liver toxicity prediction model performance across all lots for the thioacetamide treatment.

Lots	Gene Level Feature Sets		
	PTGS (All)	PTGS (Core)	TMG
A	0.75	0.71	0.68
B	0.80	0.78	0.70
C	0.94	0.89	0.83
D	0.88	0.80	0.79
E	0.85	0.81	0.74

PTGS: Predictive toxicogenomics space; TMG: toxicity module gene.

**Table 5:** Fold-change values for the key hepatocyte transporters and corresponding genes from different lots after thioacetamide exposure. The up- and down-regulated fold-change values are indicated in red and green, respectively. SD, standard deviation.

Drug Transporters	Names	Gene Symbol	A	B	C	D	E	Avg.	SD
Basolateral or sinusoidal uptake transporters	OATP1B3	*SLCO1B3*	0.37	−0.10	−0.33	−0.34	1.27	0.17	0.68
	OATP2B1	*SLCO2B1*	0.76	0.29	1.19	0.58	−2.49	0.07	1.46
	OAT2	*SLC22A7*	2.56	−1.88	−3.97	−1.59	4.25	−0.13	3.40
	OAT7	*SLC22A9*	0.08	0.67	0.86	0.45	0.14	0.44	0.33
	OCT1	*SLC22A1*	0.22	−1.52	0.23	3.32	0.21	0.49	1.75
Basolateral efflux transporters	MRP3	*ABCC3*	−0.24	0.63	1.45	−0.65	0.97	0.43	0.86
	MRP4	*ABCC4*	0.24	−1.81	−1.57	2.19	0.37	−0.11	1.63
Canalicular efflux transporters	MRP2 *	*ABCC2*	0.24	0.37	0.25	0.32	0.30	0.30	0.06
	FIC1 *	*ATP8B1*	−0.22	−0.36	−0.55	−0.49	−0.16	−0.36	0.17
	BCRP *	*ABCG2*	0.25	0.44	0.14	0.18	0.27	0.26	0.12
	Pgp (MDR1)	*ABCB1*	0.20	−0.63	0.90	0.14	5.27	1.18	2.35
	MDR3	*ABCB4*	−0.41	0.37	0.85	−2.17	−0.48	−0.37	1.15
SLC transporters		*SLC16A11*	3.94	0.64	3.61	0.66	1.85	2.14	1.58
		*SLC16A13*	0.44	0.78	0.93	1.08	0.52	0.75	0.27

The star (*) symbol entries do not show significant fold-change values but show a consistent pattern across all lots.

**Table 6:** Fold-change values for the key drug metabolism cytochrome P450 (CYP) enzymes from different lots after thioacetamide exposure. The up- and down-regulated fold-change values are indicated in red and green, respectively. SD, standard deviation.

Gene Symbol	A	B	C	D	E	Avg.	SD
*CYP2C9*	0.33	0.36	1.16	0.90	0.14	0.58	0.43
*CYP3A4*	0.51	2.11	1.22	0.86	0.35	1.01	0.70
*CYP3A5*	1.05	0.87	0.87	0.91	0.90	0.92	0.07
*CYP26A1*	−2.20	−7.22	−2.66	−3.33	−3.37	−3.75	2.00
*CYP26B1*	−2.98	−5.48	−4.07	−3.10	−6.86	−4.50	1.66
*CYP1A1*	1.02	2.02	2.86	2.89	−1.46	1.46	1.81
*CYP1A2*	−0.20	0.68	0.14	0.60	−0.12	0.22	0.40
*CYP2C19*	0.27	0.33	0.85	0.69	0.82	0.59	0.27
*CYP2C8*	−0.63	−0.29	−0.28	3.45	−0.38	0.37	1.73
*CYP2E1*	0.31	0.23	0.28	0.24	0.22	0.26	0.04

## Data Availability

Data are contained within the article and Supplementary Materials.

## Supplementary Materials

The following supporting information can be downloaded at: https://www.mdpi.com/article/10.3390/ijms25063265/s1.
