# Supplementary material for: Probing Liver Injuries Induced by Thioacetamide in Human In Vitro Pooled Hepatocyte Experiments
Source: Int J Mol Sci. 2024 Mar 13;25(6):3265. doi: 10.3390/ijms25063265 (PMC10970511; doi:10.3390/ijms25063265)
Supplement: Supplementary file 1 [file ijms-25-03265-s001.zip › 11_8_2023_SUPP_TA_FINAL.pdf]

Supplementary Material

Probing liver injury induced by thioacetamide in human *in vitro* pooled hepatocyte experiments

Himanshu Goel[1,2]*, Richard L. Printz[3,4]*, Venkat R. Pannala[1,2], Mohamed Diwan M. AbdulHameed[1,2], and Anders Wallqvist[1]*

[1]*Department of Defense Biotechnology High Performance Computing Software Applications Institute, Telemedicine and Advanced Technology Research Center, U.S. Army Medical Research and Development Command, Fort Detrick, MD, USA*

[2]*The Henry M. Jackson Foundation for the Advancement of Military Medicine, Inc., Bethesda, MD, USA*

[3]*Department of Molecular Physiology and Biophysics, Vanderbilt University School of Medicine, Nashville, TN, USA*

[4]*Division of Diabetes, Endocrinology, and Metabolism, Department of Medicine, Vanderbilt University Medical Center, Nashville, TN, USA*

***Correspondence:**
Dr. Himanshu Goel; Telephone: (301) 619-1976; E-mail: hgoel@bhsai.org
Dr. Richard L. Printz; Telephone: (615) 343-0526; E-mail: r.printz@vumc.org
Dr. Anders Wallqvist; Telephone: (301) 619-1989; E-mail: sven.a.wallqvist.civ@health.mil

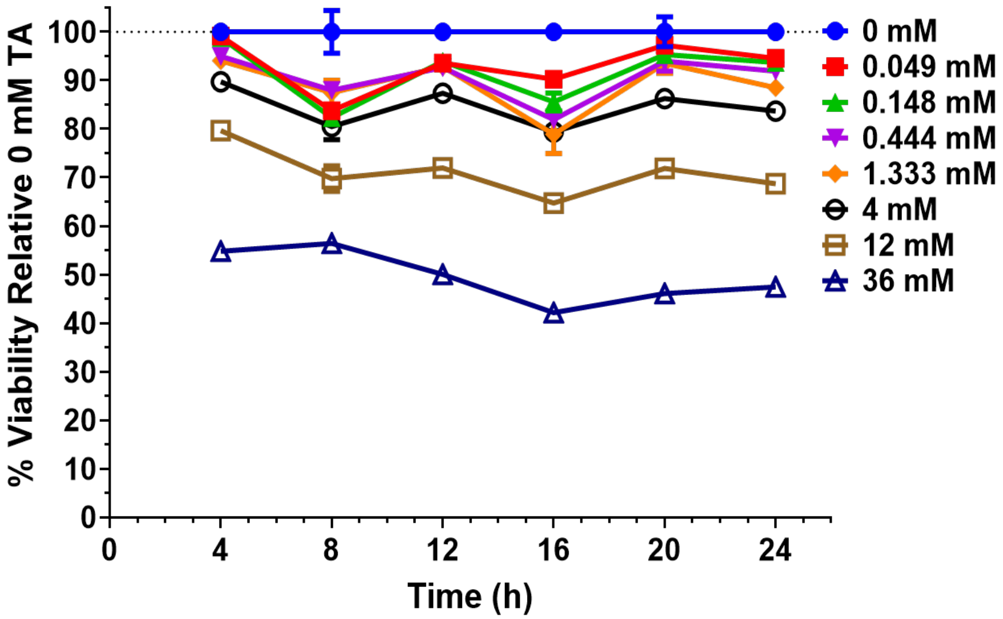

Figure S1. Time-course study to determine the length of thioacetamide (TA) exposure needed to cause detectable cell injury in pooled human hepatocytes. Hepatocytes were cultured in 96-well collagen 1-coated plates (Corning, Tewksbury, MA, USA) following the protocols and using the culture reagents provided by the supplier, Sekisui XenoTech (Kansas City, KS, USA). Cell viability was monitored by quantifying intracellular ATP levels using the CellTiter-Glo 2.0 kit (Promega, Madison, WI, USA) after exposure to various doses of thioacetamide.

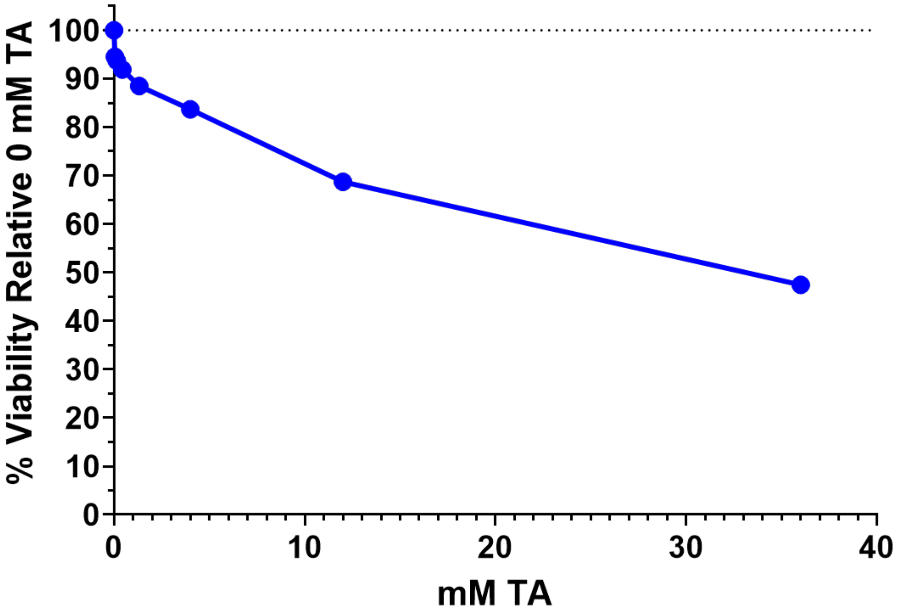

Figure S2. Analysis of pooled human hepatocyte cell viability by quantifying intracellular ATP levels after 24 hours of exposure to various doses of thioacetamide (TA).

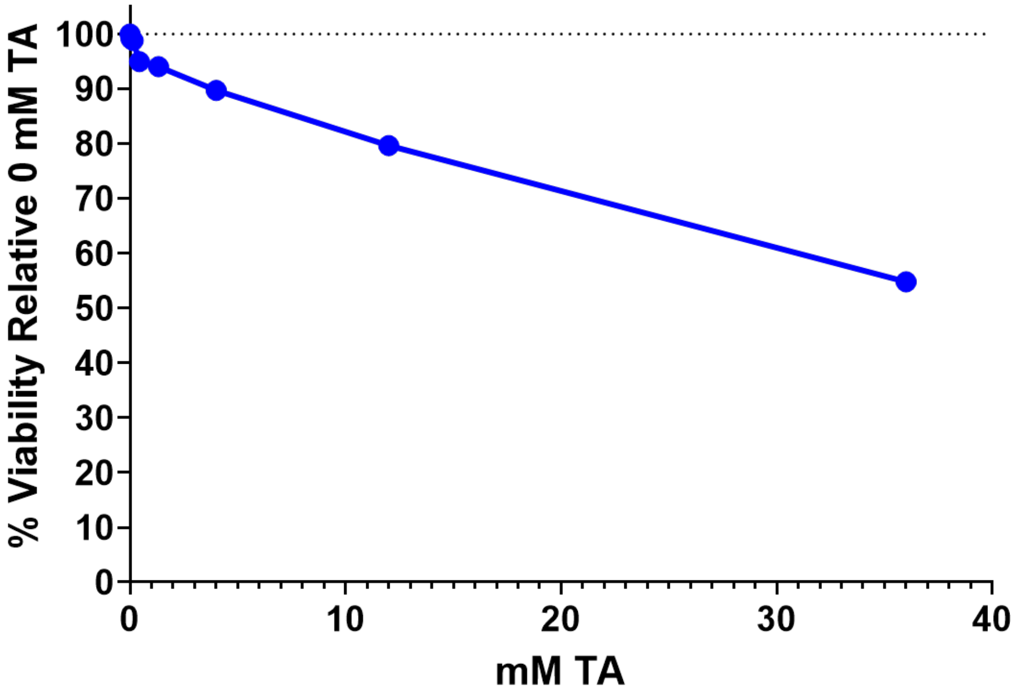

Figure S3. Analysis of pooled human hepatocyte cell viability by quantifying intracellular ATP levels after 4 hours of exposure to various doses of thioacetamide (TA).

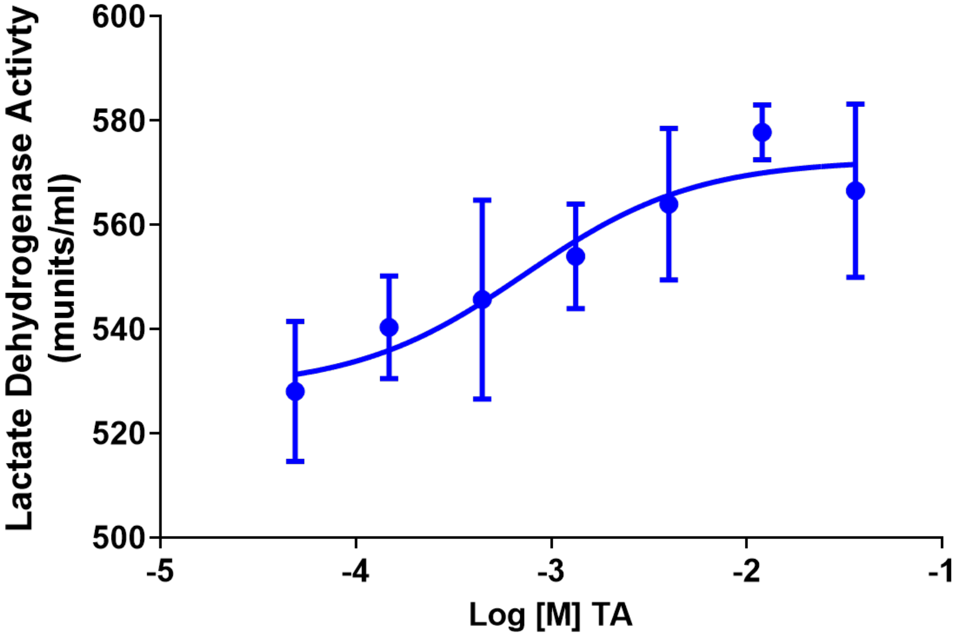

Figure S4. Analysis of pooled human hepatocyte cell viability by quantifying lactate dehydrogenase activity released into the medium as a marker of cell membrane integrity after 24

hours of exposure to various doses of thioacetamide (TA). Activity was quantified using a Lactate Dehydrogenase Activity Assay Kit (MilliporeSigma, Burlington, MA, USA).

Donor Number	Age	Gender	BMI	Race	Cause of Death	CMV (+/-)	Diabetes	Smoker (Y/N)	Lot #	Cat #
H1220	28	M	23.1	Asian	Anoxia	+	none	Y	2210146	HPCH10+
H1236	74	M	27	Caucasian	Head trauma	-	none	Y	2210146	HPCH10+
H1382	57	M	27.8	Caucasian	Anoxia	-	none	N	2210146	HPCH10+
H1401	48	M	22.1	African American	Anoxia	+	none	N	2210146	HPCH10+
H1417	46	F	37.5	African American	CVA	+	none	N	2210146	HPCH10+
H1418	51	F	30.8	African American	Anoxia	-	none	Y	2210146	HPCH10+
H1453	48	F	37.7	Caucasian	Anoxia	-	Insipidus	N	2210146	HPCH10+
H1460	40	F	38.1	Caucasian	Anoxia	-	none	N	2210146	HPCH10+
H1467	52	F	31.1	Caucasian	Anoxia	-	none	N	2210146	HPCH10+
H1474	64	M	19.4	Asian	CVA	+	none	N	2210146	HPCH10+
H1428	53	F	28.6	Caucasian	Anoxia	-	none	Y	2010236	HPCH10+
H1439	67	F	20.5	Caucasian	CVA	+	none	N	2010236	HPCH10+
H1441	37	M	26.4	Caucasian	Anoxia	-	none	N	2010236	HPCH10+
H1442	66	F	21.8	Caucasian	Head trauma	-	none	N	2010236	HPCH10+
H1443	53	M	37.9	Caucasian	CVA	-	none	N	2010236	HPCH10+
H1444	50	F	22.8	Caucasian	Head trauma	+	none	Y	2010236	HPCH10+
H1445	16	F	23.8	Caucasian	CVA	+	none	N	2010236	HPCH10+
H1447	10	M	22.2	Hispanic	Head trauma	+	none	N	2010236	HPCH10+
H1448	66	M	40	Caucasian	Head trauma	+	none	N	2010236	HPCH10+
H1449	61	M	34.8	Caucasian	Head trauma	+	none	N	2010236	HPCH10+
H1205	57	M	23.5	Caucasian	CVA	+	none	N	1810050	HPCH10+
H1211	9	F	18.6	Caucasian	Anoxia	-	none	N	1810050	HPCH10+
H1254	62	F	32.8	Caucasian	CVA	-	none	N	1810050	HPCH10+
H1295	45	M	29.8	Caucasian	Anoxia	-	none	N	1810050	HPCH10+
H1296	46	M	21.1	Caucasian	Anoxia	-	none	N	1810050	HPCH10+
H1308	32	F	32.2	Caucasian	Anoxia	-	type 2	N	1810050	HPCH10+
H1329	42	M	29	Caucasian	Head trauma	+	none	N	1810050	HPCH10+
H1358	57	F	29	Caucasian	CVA	-	none	N	1810050	HPCH10+
H1361	46	M	31.3	Caucasian	Anoxia	+	none	N	1810050	HPCH10+
H1366	55	F	32	Caucasian	CVA	+	none	Y	1810050	HPCH10+
H1207	42	F	25	Asian	CVA	+	none	N	1810126	HPCH05+
H1219	24	F	26.5	Caucasian	Trauma	+	none	Y	1810126	HPCH05+
H1229	41	M	34.2	African American	CVA	+	unknown	N	1810126	HPCH05+
H1252	57	F	29.7	Caucasian	CVA	+	none	Y	1810126	HPCH05+
H1371	26	M	26.1	Caucasian	Head trauma	-	none	N	1810126	HPCH05+
H1408	19	F	29.5	Caucasian	Head trauma	+	none	N	2110283	HPCH05+
H1419	21	M	20.7	Caucasian	Head trauma	+	none	N	2110283	HPCH05+
H1442	66	F	21.8	Caucasian	Head trauma	-	none	N	2110283	HPCH05+
H1462	59	F	19.4	Caucasian	Anoxia	+	type 2	N	2110283	HPCH05+
H1463	57	M	33.7	Caucasian	CVA	-	none	N	2110283	HPCH05+
**AVG Age**	**46.3**	**40 T**	**28.0**	**4 African American**	**15 Anoxia**	**21 +**	**36 None**	**8 Y**		
**AVG Age F**	**46.2**	**21 F**	**28.1**	**3 Asian**	**13 CVA**	**19 -**	**1 Insipidus**	**32 N**		
**AVG Age M**	**46.3**	**19 M**	**27.9**	**1 Hispanic**	**11 Head trauma**		**2 Type 2**			
				**32 Caucasian**	**1 Trauma**		**1 Unknown**			

Figure S5. Donor demographics for the pooled primary human hepatocytes.
